# Efficacy and safety of subcutaneous injection of botulinum toxin in the treatment of Chinese postherpetic neuralgia compared to analgesics: a systematic review of randomized controlled trials and meta-analysis

**DOI:** 10.3389/fneur.2024.1479931

**Published:** 2024-10-17

**Authors:** Hui Wang, Ping Lin

**Affiliations:** Department of Geriatrics, Hangzhou Third People’s Hospital, Hangzhou, Zhejiang, China

**Keywords:** postherpetic neuralgia, botulinum toxin, systematic review, meta-analysis, botulinum toxin A

## Abstract

**Objective:**

The purpose of this meta-analysis is to investigate the efficacy and safety of a subcutaneous injection of botulinum toxin in the treatment of postherpetic neuralgia (PHN) compared to analgesics.

**Methods:**

We searched PubMed, Cochrane Library, Embase, Web of Science, Chinese National Knowledge Infrastructure (CNKI), and Wanfang for randomized controlled trials (RCTs) from inception to 10 September 2023. The primary clinical outcomes included visual analog scale (VAS) pain scores and clinical effective rates. The secondary clinical outcome included the adverse event rate during follow-up.

**Results:**

A total of 14 studies with 1,358 participants were included in the meta-analysis. Among the included patients, 670 participants received botulinum toxin A injections and 688 participants received other medication treatments. The botulinum toxin-A (BTX-A) group exhibited lower pain scores [week 2: Mean difference (MD): −1.91, 95% confidence interval (CI): −2.63 to −1.20, and *p* < 0.00001; week 4: MD: –1.69, 95% CI: −2.69 to −0.68, and *p* < 0.00001; week 8: MD: –1.66, 95% CI: −2.20 to −1.12, and *p* < 0.00001; week 12:MD: –1.83, 95% CI: −2.70 to −0.96, and *p* < 0.00001; and week 24: MD: -1.07, 95% CI: −1.16 to −0.99, and *p* < 0.00001]. The effective rate was significantly higher in patients who received BTX-A for postherpetic neuralgia compared to those who received lidocaine or gabapentin (lidocaine: MD: –1.55, 95% CI: −2.84 to −0.27, and *p* = 0.02 and gabapentin: MD: –1.57, 95% CI: −2.12 to −1.02; and *p* < 0.00001). There was no difference in the incidence of adverse events between the treatment groups [odds ratio (OR): 1.25, 95% CI: 0.43 to 3.61, and *p* = 0.69].

**Conclusion:**

Our meta-analysis showed that BTX-A has certain advantages in relieving postherpetic neuralgia compared to analgesics. In addition, BTX-A is safe for treating postherpetic neuralgia, with no notable side effects.

**Systematic review registration:**

https://www.crd.york.ac.uk/prospero/, identifier CRD42021289813.

## Introduction

Postherpetic neuralgia (PHN) is defined as local neuropathic pain that persists for more than 3 months following the initial acute zoster infection (1). Its clinical manifestations include continuous burning pain, as well as sensations of shooting, stabbing, and tactile pain, similar to electric shocks. Research indicates that the incidence rate of PHN ranges from 5 to 30%, with a discernible correlation between advancing age and diminishing immunity (2). Moreover, due to low immunity levels in elderly patients, PHN is difficult to cure completely, and the pain they experience can be intense, persistent, and often intolerable (3). In some cases, individuals may endure uncontrolled pain for over a decade. Long-term pain not only causes immense physical distress but also has emotional repercussions, potentially leading to depression. This multifaceted impact adversely affects both the overall quality of life and the daily functioning of these patients (4, 5).

Currently, drugs used in the treatment of PHN in clinical practice mainly include tricyclic antidepressants, gabapentin, pregabalin, and opioids (6). Although analgesics can partially alleviate PNH, the long-term clinical efficacy of its treatment is still uncertain due to the risk of liver and kidney damage (7).

Botulinum toxin-A (BTX-A), a potent neurotoxin produced by *Clostridium botulinum*, inhibits acetylcholine release at neuromuscular junctions, causing muscle relaxation (8). It is currently used in clinical practice to treat chronic pain, although the specific mechanism of its action is not fully understood. It is posited that it may block the release of calcitonin gene-related peptides and other neuropeptides by inhibiting the release of pain mediators in motor and sensory neurons (9). In addition, it could suppress the release of sensory inflammatory mediators and peripheral neurotransmitters and inactivate sodium channels in the central neuronal membrane (10, 11).

Currently, studies have shown that BTX-A has a certain effect on the treatment of various types of neuralgia, such as herpes zoster neuralgia, trigeminal neuralgia (TN), and diabetes neuropathy (12).

However, specific research on the effectiveness and safety of BTX-A in treating PHN is relatively limited compared to analgesics.

Therefore, we performed a meta-analysis of randomized controlled trials (RCTs) to investigate the efficacy and safety of BTX-A compared to analgesics in the treatment of PHN and to provide clinical guidance for treatment options available to these patients.

## Methods

This research was conducted according to the Preferred Reporting Items for Systematic reviews and Meta-Analyses (PRISMA) guidelines (13). The protocol for this review was registered in PROSPERO under the identification number CRD42021289813.

### Search strategy

In this meta-analysis, we conducted a comprehensive search of PubMed, Embase, the Cochrane Library, Web of Science, Chinese National Knowledge Infrastructure (CNKI), and Wanfang databases for randomized controlled trials from their inception up to 10 September 2023. The detailed search terms and strategy are available in Supplementary material 1. In addition, the reference lists of the retrieved articles were manually and independently screened to identify potentially relevant articles. The inclusion criteria were explicitly defined, and no discrepancies in the search results were found.

### Selection criteria

In this systematic review, we applied the following inclusion criteria: (1) randomized controlled clinical trials (RCTs); (2) patients clinically diagnosed with postherpetic neuralgia based on the American Academy of Neurology 2004 or Chinese Medical Association criteria; and (3) interventions including BTX-A, administered either alone or in combination with the same active treatments used in the control group. The exclusion criteria included the following: (1) animal experiments; (2) systematic reviews, meta-analyses, and non-systematic reviews; (3) case reports; and (4) studies not reporting the use of BTX in the treatment of PHN. The selection process was meticulous, ensuring a rigorous and reliable inclusion of studies in the meta-analysis.

### Main outcome variables

Two authors (HW and PL) independently extracted the following details from each study: (1) Basic information (author, publication year, sample size, and follow-up duration); (2) participant Characteristics (age, gender, and average disease duration); (3) intervention and comparison (parameters of BTX-A and control group dosages); and (4) primary clinical outcomes, including visual analog scale (VAS) pain scores and clinical effective rates. The VAS is a standardized tool for assessing pain. Pain intensity is categorized on a scale from 0 to 10, where 0 represents no pain; scores of 1–3 indicate mild, tolerable pain; scores of 4 to 6 indicate moderate pain that disrupts sleep but remains tolerable; and scores of 7–10 indicate severe pain that is intolerable and affects both sleep and appetite. The secondary clinical outcome is the adverse event rate during follow-up. Any discrepancies between the reviewers regarding data extraction were resolved through discussion until a consensus was reached.

### Risk of bias assessment

Two authors (HW and PL) independently assessed the risk of bias in the included studies using the Cochrane risk-of-bias tool for randomized trials, which evaluates the following domains: random sequence generation, allocation concealment, the blinding of participants and personnel, the blinding of outcome assessment, incomplete outcome data, selective reporting, and other forms of bias. In each domain, the risk of bias was discerned and categorized as ‘high’, ‘unclear,’ or ‘low’ (14). Disagreements between the review authors regarding the assessment of study quality were resolved through discussion until a consensus was reached.

### Statistical analysis

Data were analyzed using Review Manager 5.4 (Cochrane Collaboration) and Stata 16. All the studies were grouped and analyzed considering the outcome variable of each study and the characteristics of the intervention. Mean differences (MDs) with 95% confidence intervals (CIs) were calculated for continuous variables (VAS pain score), and odds ratios (ORs) with 95% CIs were calculated for dichotomous variables (effective rate and adverse event rate). Heterogeneity was evaluated using the *I*^2^ test. When *I*^2^ was <50%, a fixed-effects model was adopted. When *I*^2^ was >50%, we used a random-effects model. In addition, funnel plots were used initially to evaluate visual publication bias, while Begg’s regression test was used to inferentially evaluate publication bias. Statistical significance was set at a *p*-value of <0.05.

## Results

### Study selection

**Figure 1:**
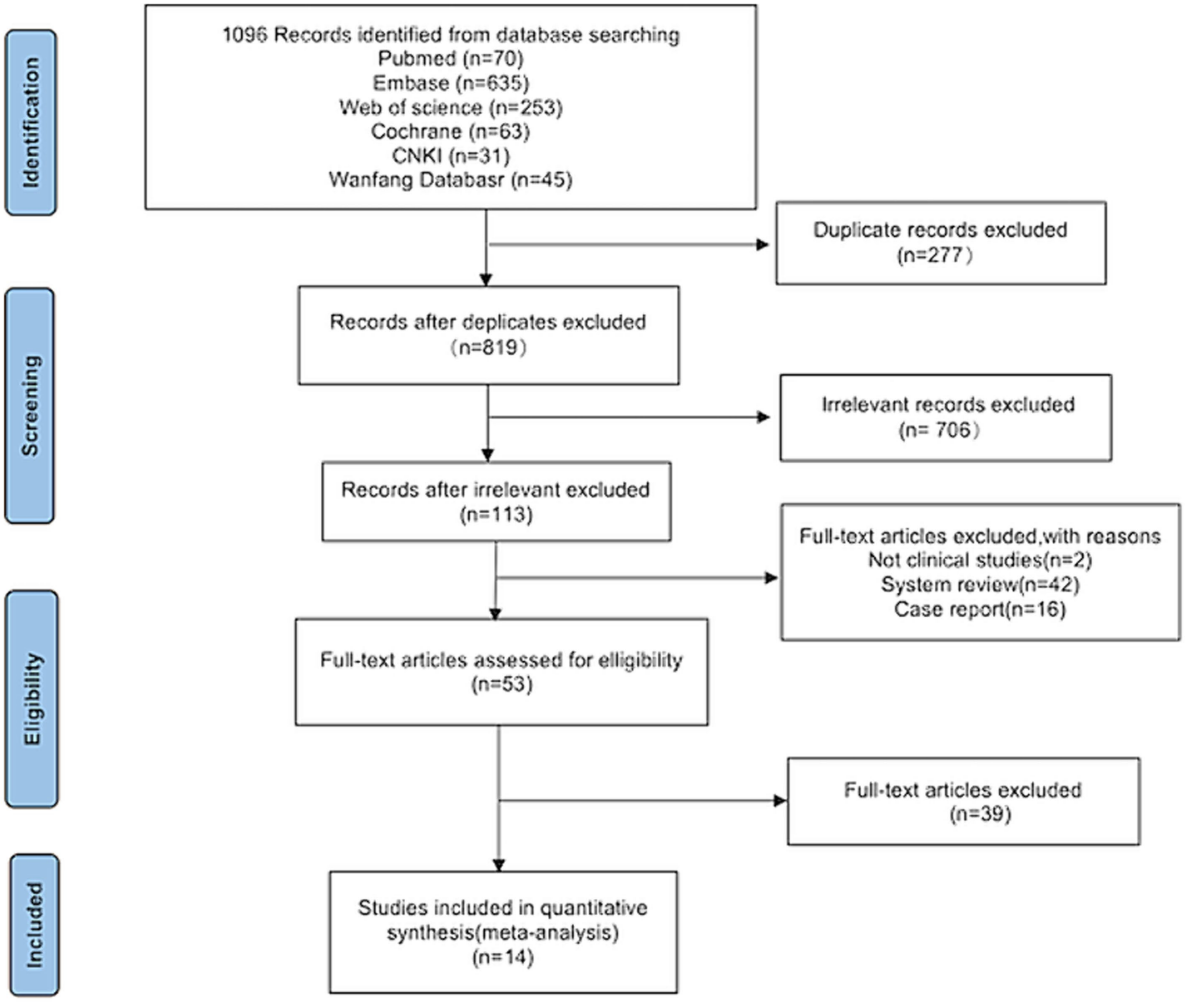
PRISMA flowchart of the study selection.

Following a search of the six databases, 1,096 articles related to the topic were identified. After screening titles and abstracts, 277 duplicates and 706 irrelevant articles were excluded, leaving 113 articles that were deemed potentially eligible for inclusion. After further analysis of the full texts, 60 studies were excluded. Among these, two were not clinical studies, 42 were system reviews, and 16 were case reports. Finally, 14 studies (15–28) were included in the meta-analysis (Figure 1).

### Study characteristics

Our study encompassed publications from 2013 to 2023, involving a total of 1,358 participants. All the participants were diagnosed with postherpetic neuralgia. A total of 12 studies (15–20, 22–27) included patients who were diagnosed based on the Chinese Medical Association criteria, while 2 studies (21, 28) included patients who were diagnosed based on the AAN 2004 criteria. All included patients were from China. A total of 670 participants received BTX-A injections, and 688 participants received analgesic medication treatments. BTX-A was sourced from the China Biologic Products Institute (Lanzhou Branch), with a maximum total dosage of 100 units. Although a wide range of dosages were used and different techniques were employed, including intracutaneous and/or subcutaneous injections, the positive effect of BTX-A against PHN was observed in all patients, albeit with varying individual effect sizes. Seven studies (15, 16, 18, 20–22, 26) reported side effects. The detailed characteristics of the studies are presented in Table 1.

**Table 1:** Characteristics of the studies included in the meta-analysis.

Study	Study region	Diagnostic criteria	Number of cases		Gender (man/woman)		Mean age (year)		Follow-up time	Duration of disease (m)		Interventions					Outcome measures
			Experimental group	Control group	Experimental group	Control group	Experimental group	Control group		Experimental group	Control group	Experimental group				Control group	
												Intervention drugs	Injection site	Frequency of injection	Doses		
Dai YE (2018) (16)	China	the CMA criteria	39	32	21/18	18/14	66.2 ± 8.4	64.5 ± 8.9	12 months	2.19 ± 0.40	2.13 ± 0.32	BTX-A (Chi-Botox, s.c.) + Gabapentin	pain area	9	2.5-5u at each point, 100 U	Gabapentin +0.8%Lidocaine (20 mL, s.c.)	VAS, The severity of pain symptom, Adverse reactions
Gan WQ (2014) (17)	China	NA	31	31	not applicable	not applicable	not applicable	not applicable	12 weeks	not applicable	not applicable	BTX-A (Chi-Botox, s.c.) + Gabapentin	skin lesion area	1	Every 1.5–2 cm, 100 U	Gabapentin	VAS, QS, Adverse reactions
Liu HP (2009) (18)	China	the CMA criteria	30	30	14/16	12/18	56.80 ± 8.18	56.36 ± 7.2	24 weeks	2.10 ± 0.66	2.03 ± 0.79	BTX-A (Chi-Botox, s.c.)	pain area	1	2.5-5u at each point, 100 U	Carbamazepine (100 mg/day)	VAS, McGill pain questionnaire, effective rate of treatment
Pan WJ (2020) (19)	China	the CMA criteria	55	55	27/28	29/26	57.5 ± 10.7	56.6 ± 10.5	8 weeks	10.66 ± 3.28	10.34 ± 3.13	BTX-A (Chi-Botox, s.c.) + Pregabalin	pain area	1	2u at each point, 100 U	Pregabalin (150 mg, bid)	VAS, effective rate of treatment, Adverse reactions
Peng T (2022) (20)	China	the CMA criteria	20	20	8/12	10/10	62-84 years	60-85 years	16 weeks	1.25–11.5	1.25–12	BTX-A (Chi-Botox, s.c.)	pain area	1	5u at each point, 50-100 U	Gabapentin (0.3 g, tid)	VAS
Wu HL (2021) (21)	China	the CMA criteria	22	22	13/9	12/10	73.64 ± 5.26	73.60 ± 8.78	12 weeks	11.09 ± 8.14	10.91 ± 8.99	BTX-A (Chi-Botox, i.c.) + Pregabalin	pain area	1	Every 1–1.5 cm, 50–100 U	Pregabalin (150 mg, bid)	VAS, effective rate of treatment, Adverse reactions
Xue RL (2017) (22)	China	the AAN 2004 criteria	40	40	15/15	16/14	53.78 ± 6.34	53.37 ± 6.28	8 weeks	2.6 ± 0.65	2.5 ± 0.33	BTX-A (Chi-Botox, s.c.)	pain area	1	100 U	Lidocaine (100 mg, s.c.)	VAS, effective rate of treatment, Adverse reactions
Xu XR (2021) (23)	China	the CMA criteria	30	30	16/14	18/12	53.47 ± 8.52	52.14 ± 8.67	8 weeks	1.95 ± 0.68	1.89 ± 0.65	BTX-A (Chi-Botox, i.c.) + Gabapentin	pain area	6	2.5u at each point, 100 U	Gabapentin (0.3 g, tid)	VAS, PSQI, effective rate of treatment, Adverse reactions
Yang F (2014) (24)	China	NA	200	200	115/85	120/80	56.34 ± 4.88	56.32 ± 5.69	8 weeks	2.06 ± 0.66	2.04 ± 0.17	BTX-A (Chi-Botox, s.c.)	skin lesion area	6	2.5-5u at each point, 100 U	2%Lidocaine (5 mL, s.c.)	VAS, effective rate of treatment
Yang YP (2022) (25)	China	NA	72	72	42/30	40/32	53.25 ± 3.15	53.31 ± 3.16	24 weeks	10.15 ± 1.65	10.21 ± 1.71	BTX-A (Chi-Botox, s.c.)	pain area	9	0.25u at each point, 100 U	Triamcinolone acetonide (1 mL, s.c.) + Lidocaine (1 mL, 30 mg)	VAS, NPS, Frequency and duration of pain attacks
Yang YZ (2015) (26)	China	the CMA criteria	40	40	24/16	23/17	65.3 ± 6.7	65.1 ± 6.9	12 weeks	24.8 ± 2.7	24.5 ± 2.5	BTX-A (Chi-Botox, s.c.) + Gabapentin	pain area	1	2.5u at each point, 100 U	Gabapentin (0.9 g/day)	VAS
Zhai Y (2021) (27)	China	the CMA criteria	40	40	20/20	22/18	59.75 ± 4.85	59.85 ± 5.65	24 weeks	not applicable	not applicable	BTX-A (Chi-Botox, s.c.)	pain area	1	2.5u at each point, 100 U	Lidocaine (1 mL, 30 mg, s.c.)	VAS, Adverse reactions
Zhong GM (2021) (28)	China	the CMA criteria	31	31	17/14	18/13	63.94 ± 2.37	63.48 ± 2.45	8 weeks	not applicable	not applicable	BTX-A (Chi-Botox, s.c.)	pain area	1	0.25u at each point, 100 U	Triamcinolone acetonide (15 mg, s.c.) +2%Lidocaine (5 mL, s.c.)	VAS, effective rate of treatment
Zhu MM (2018) (29)	China	the AAN 2004 criteria	38	27	not applicable	not applicable	not applicable	not applicable	8 weeks	not applicable	not applicable	BTX-A (Chi-Botox, s.c.)	pain area	1	2.5u at each point, 100 U	Triamcinolone acetonide (1 mL, s.c.) + Lidocaine (1 mL,30 mg)	VAS, effective rate of treatment

The CMA criteria, the Chinese Medical Association criteria; the AAN 2004 criteria, the American Academy of Neurology criteria; Chi-Botox, Chinese Botox; VAS, visual analog scale; BTX-A, botulinum toxin A; s.c., subcutaneous; i.c., intracutaneous; bid, twice a day; and tid, three times a day.

### Quality assessment

**Figure 2:**
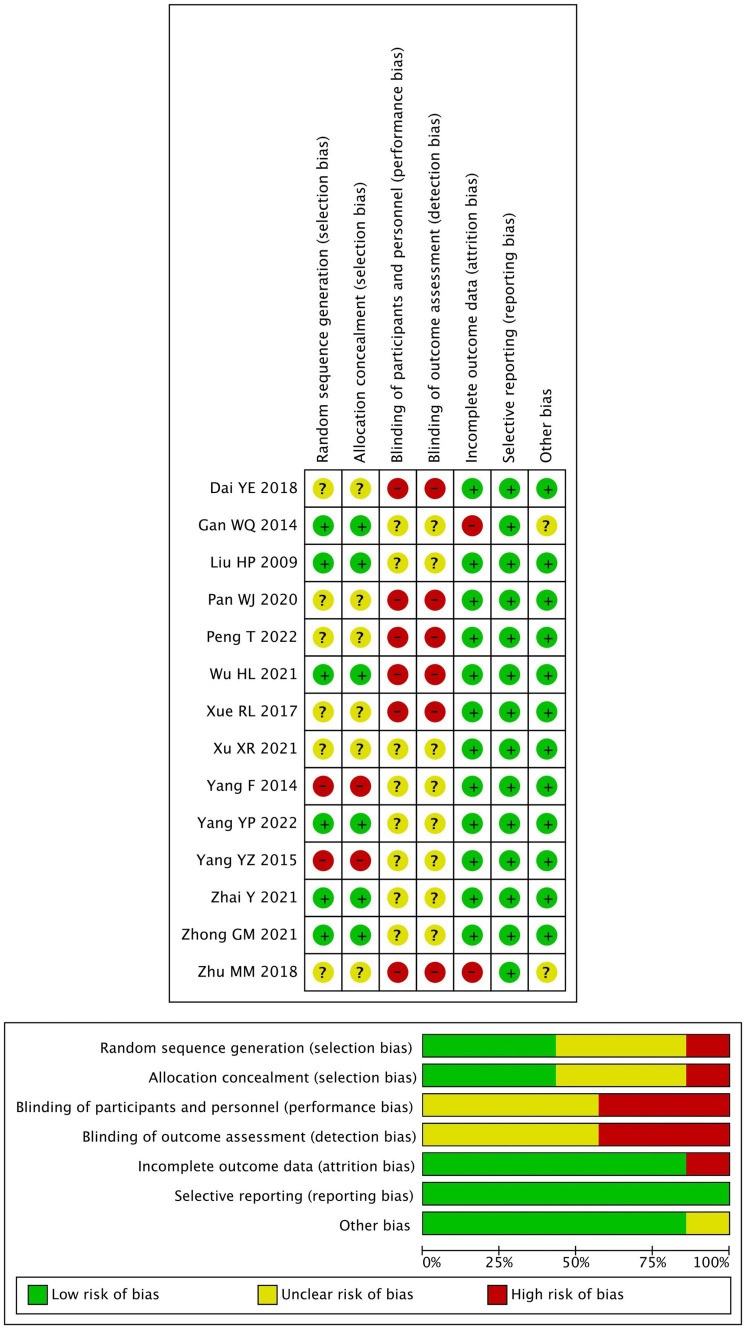
Risk of bias of the included studies.

The quality assessment of the included studies is shown in Figure 2.

All included trials were single-center randomized studies. Seven studies reported using a random number method for grouping, while the remaining seven studies did not specify their randomization methods. In addition, the blinding of participants and personnel was unclear in all studies as insufficient information provided.

### Result analysis

We investigated the effectiveness and safety of subcutaneous injections of BTX-A in treating postherpetic neuralgia compared to analgesics by analyzing VAS pain scores, clinical efficacy, and the incidence of adverse events.

#### VAS scores

**Figure 3:**
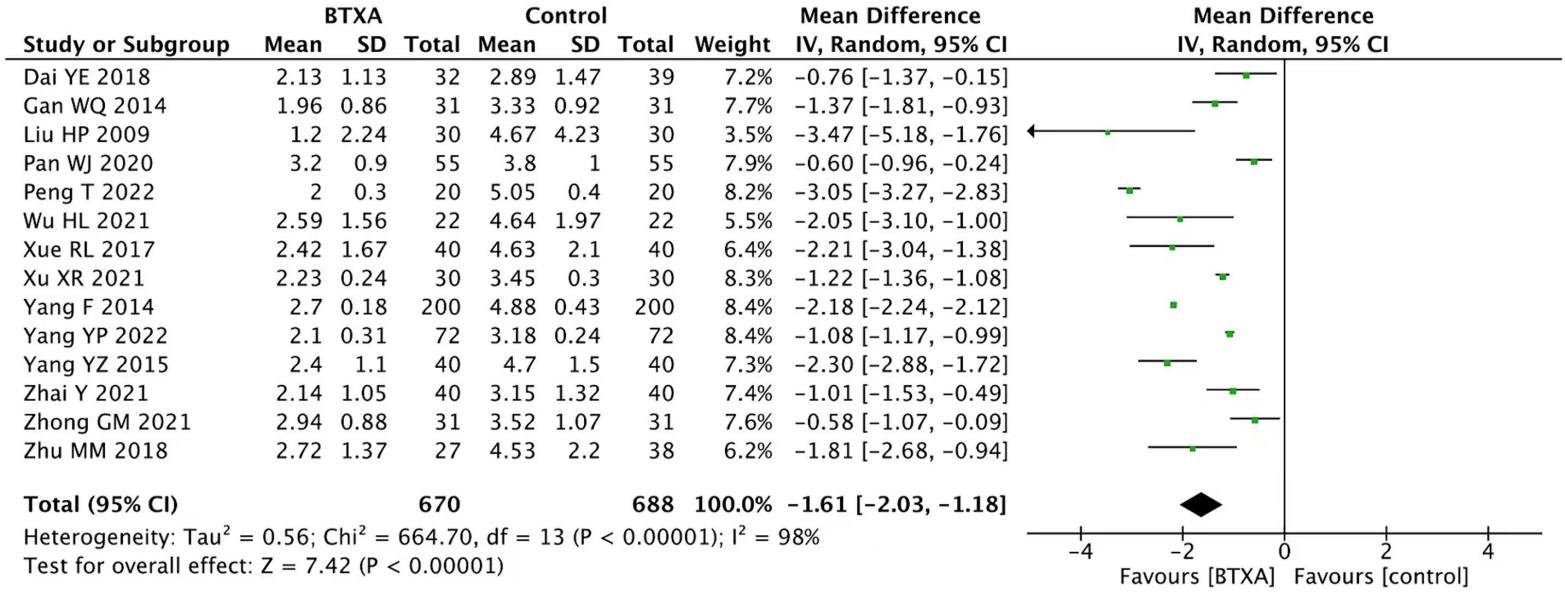
Comparison of the VAS scores between BTX-A and control treatment for PNH at the end of follow-up.

We conducted a summary analysis of the 14 studies (15–28), evaluating VAS at the end of follow-up. The results showed that BTX-A was superior to other drug treatments in relieving herpes zoster neuralgia (MD: –1.61, 95% CI: −2.03 to −1.18, and *p <* 0.00001; Figure 3).

**Figure 4:**
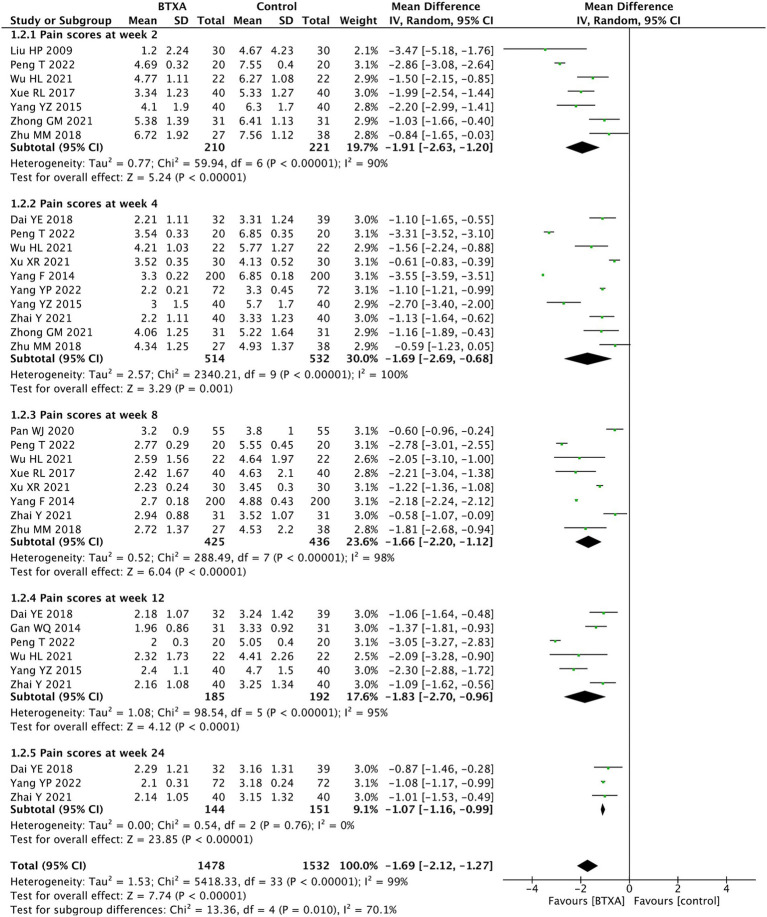
Comparison of the VAS scores between BTX-A and control therapy for PNH at different follow-up time points.

We conducted a subgroup analysis of the VAS scores at different follow-up time points. The results showed that BTX-A was superior to analgesics treatments in relieving herpes zoster neuralgia at 2, 4, 8, 12, and 24 weeks after intervention (week 2: MD: –1.91, 95% CI: −2.63 to −1.20, and *p* < 0.00001; week 4: MD: –1.69, 95% CI: −2.69 to −0.68, *p* < 0.00001; week 8: MD: –1.66, 95% CI: −2.20 to −1.12, and *p* < 0.00001; week 12:MD: –1.83, 95% CI: −2.70 to −0.96, and *p* < 0.00001; and week 24: MD: –1.07, 95% CI: −1.16 to −0.99, and *p* < 0.00001, Figure 4).

**Figure 5:**
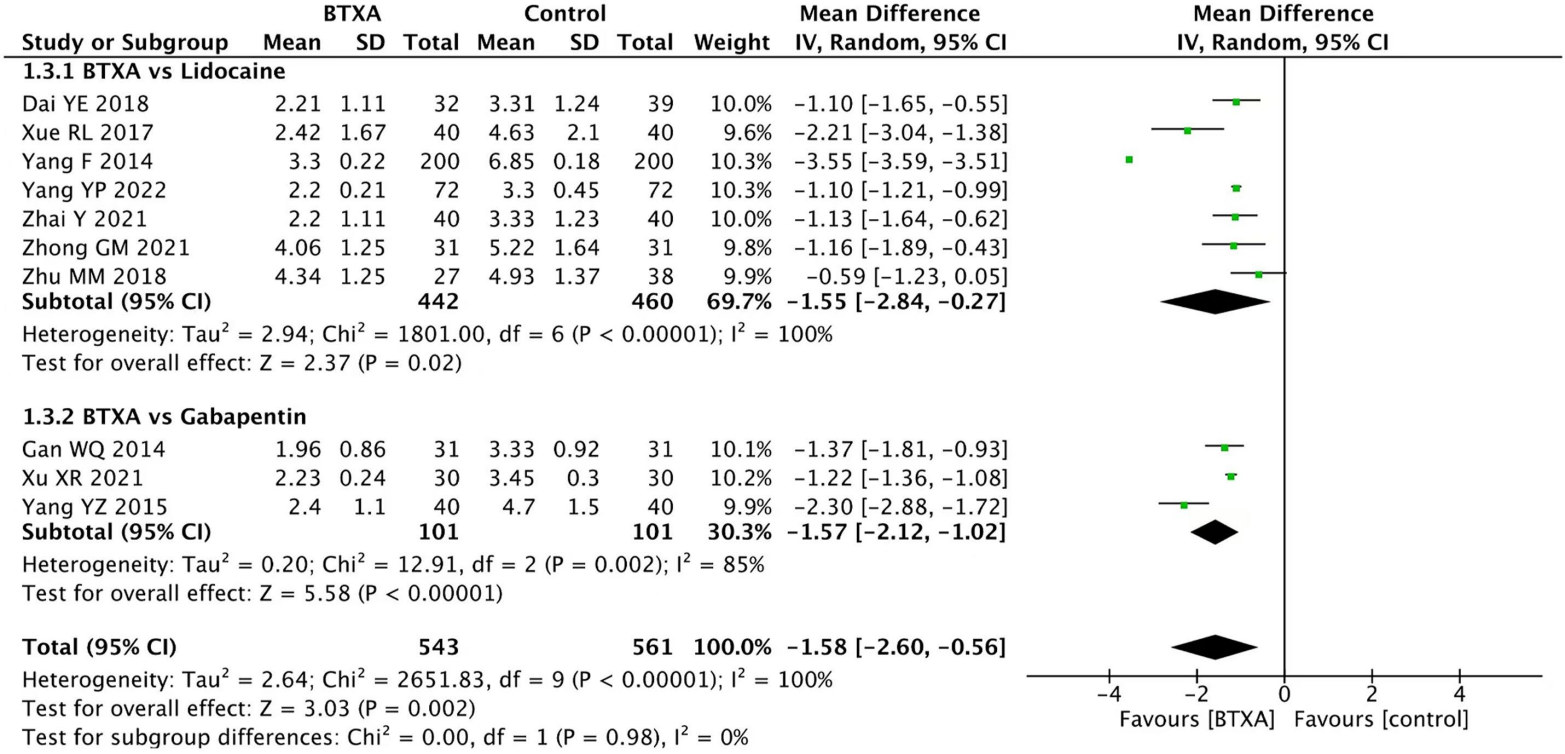
Comparison of the VAS scores between the BTX-A and different control groups in the treatment of PNH.

In addition, we conducted a subgroup analysis of different drug treatments in the control group. Among the included studies, seven trials (15, 21, 23, 24, 26–28) compared BTX-A with lidocaine treatment, with 442 patients receiving BTX-A treatment and 460 receiving lidocaine. Three trials (16, 22, 25) compared BTX-A with gabapentin treatment, with 101 patients receiving BTX-A treatment and 101 receiving gabapentin treatment. The results showed that BTX-A was superior to both lidocaine or gabapentin in treating herpes zoster neuralgia (lidocaine: MD: –1.55, 95% CI: −2.84 to −0.27, and *p* = 0.02; gabapentin: MD: –1.57, 95% CI: −2.12 to –1.02, and *p* < 0.00001, Figure 5).

**Figure 6:**
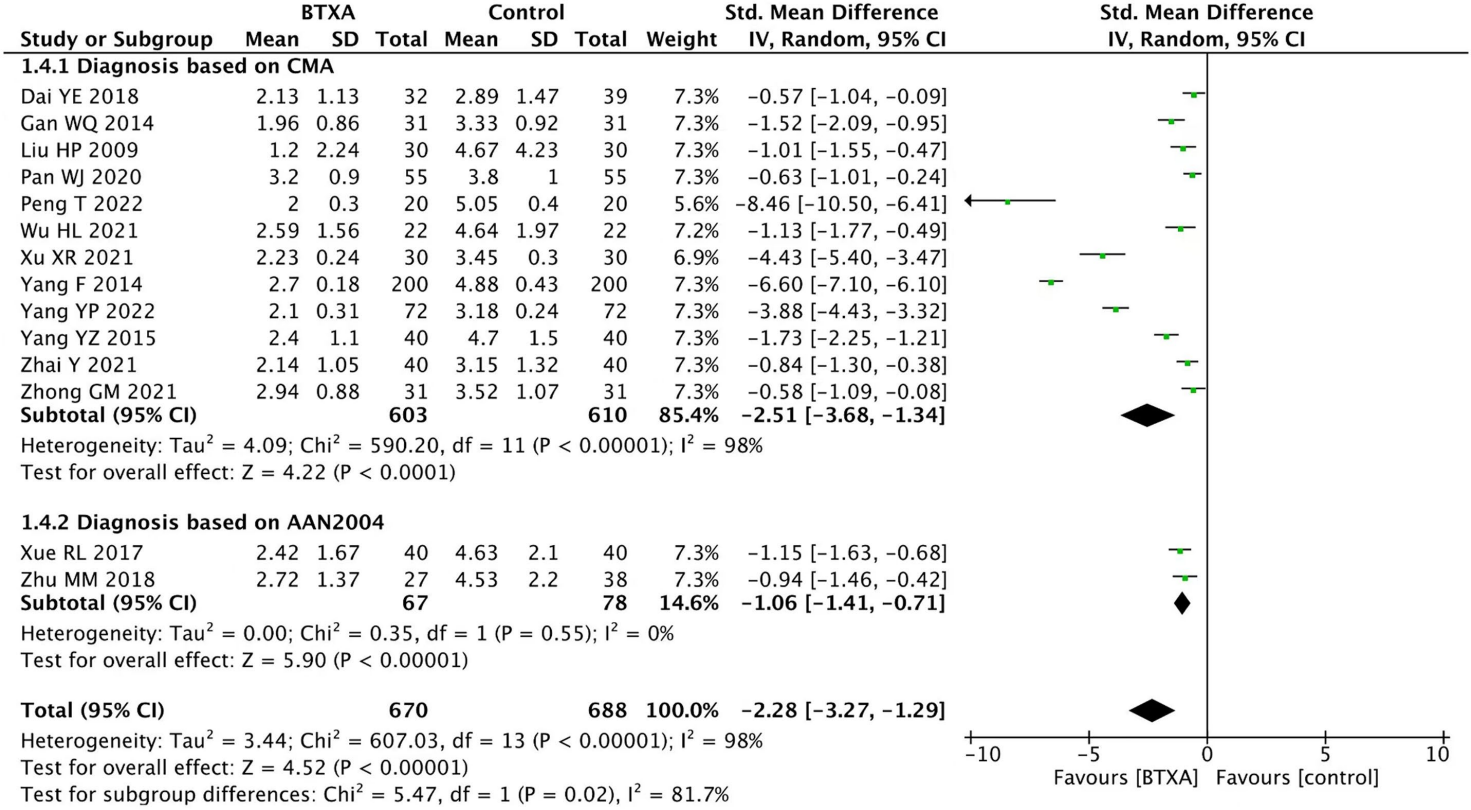
Comparison of the VAS scores between BTX-A and control therapy for PNH under different diagnostic criteria.

We conducted subgroup analysis on different diagnostic criteria for herpes zoster neuralgia. Among the included studies, 12 studies (15–20, 22–27) used the Chinese Medical Association criteria and two studies (21, 28) used the AAN 2004 criteria. The analysis results showed that under different diagnostic criteria, BTX-A was superior to other analgesic treatments in relieving herpes zoster neuralgia (Figure 6).

#### Effective rate

**Figure 7:**
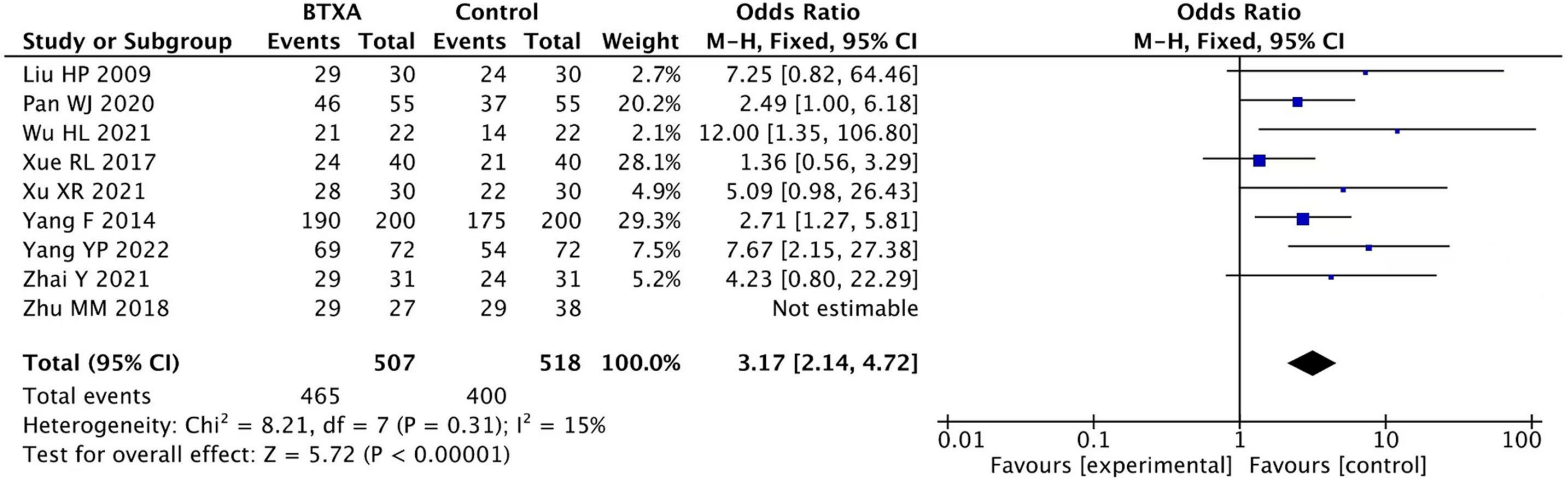
Comparison of the effective rates of BTX-A and control therapy for PNH.

Nine trials (17, 18, 20–24, 27, 28) reported the clinical effect rate of BTX-A for the treatment of PHN, involving 507 patients who received BTX-A treatment and 518 patients who received analgesic treatments. Pain remission was defined as a reduction in the VAS scores of over 50% at the end of the follow-up period. Our meta-analysis showed a significantly higher effective rate in the patients treated with BTX-A compared to those who received analgesics (OR: 3.17; 95% CI: 2.14 to 4.72; *p* < 0.00001; Figure 7).

#### Adverse event rate

**Figure 8:**
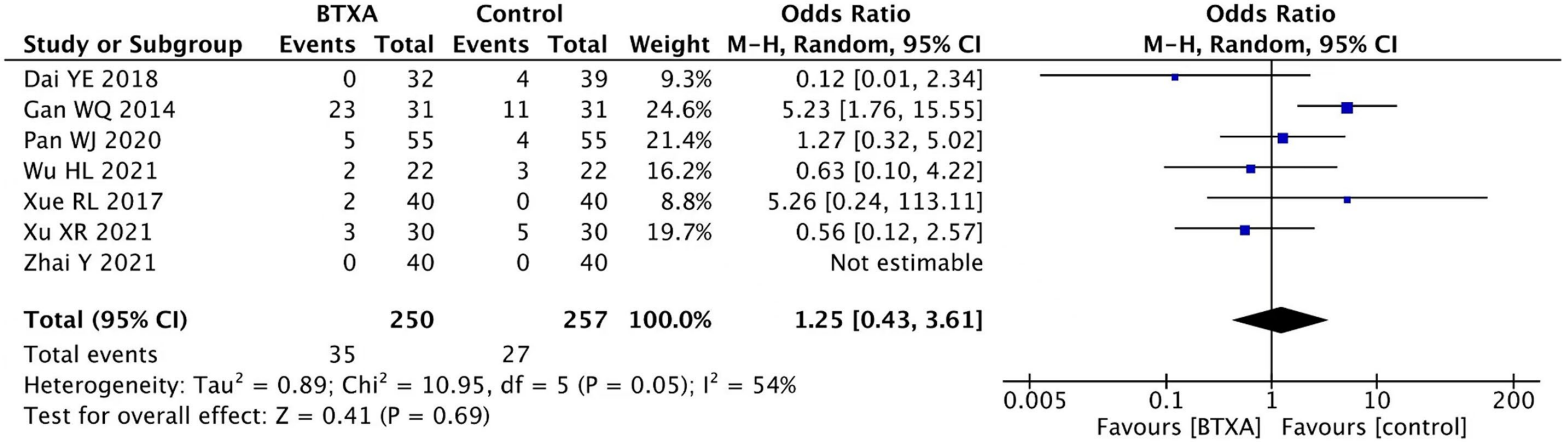
Comparison of the adverse reactions between BTX-A and control treatment for PNH.

Seven trials (15, 16, 18, 19, 21, 22, 26) reported adverse events associated with BTX-A for the treatment of PHN during the follow-up period. A total of 250 patients received treatment with BTX-A, with a total of 35 cases experiencing adverse reactions, accounting for 14%. Meanwhile, 207 patients received treatment with other analgesics, with 27 cases experiencing adverse reactions, accounting for 10.5%. None of the trials reported severe complications such as nerve damage or intracranial infection. The observed adverse events included mild pain, dizziness, drowsiness, nausea, peripheral edema, ataxia, facial erythema, and slight muscle relaxation at the injection site. Our meta-analysis showed no significant difference in the adverse event rate between the patients treated with BTX-A and those who received analgesics (OR 1.25, 95% CI 0.43 to 3.61, *p* = 0.69, Figure 8).

#### Publication bias

Publication bias is a potential concern in meta-analyses when interpreting the results. In this study, funnel plots and Begg’s tests were used to assess publication bias. Asymmetry was observed through visual inspection of the funnel plots, and the Begg’s tests also revealed statistically significant publication bias (*p* = 0.0215). The primary sources of this bias were likely the small and variable sample sizes of the randomized controlled trials, along with inherent variability in other aspects of these trials.

## Discussion

We conducted a comprehensive analysis of 14 randomized controlled studies involving VAS pain scores and effective rates. The results showed that BTX-A was superior to analgesics such as lidocaine, pregabalin, and gabapentin in relieving PHN. Furthermore, there was no difference in the incidence of adverse events between the BTX-A treatment group and the control group, and subcutaneous injections of BTX-A were not associated with any serious adverse events.

PHN is a chronic, reflex neuropathic pain syndrome, which is one of the most common sequelae of herpes zoster (29). It causes severe pain and is prone to recurrent episodes. Prolonged pain can lead to anxiety, depression, and sleep disorders, which further exacerbate the pain and significantly impact physical and mental health, as well as quality of life (30). In clinical guidelines, PHN can be treated through both drug and non-drug therapies. Commonly used treatment drugs include pregabalin, gabapentin, and tricyclic antidepressants. Non-pharmacological treatments encompass surgical interventions (such as epidural block, sympathetic nerve block, pulsed radiofrequency, and spinal cord stimulation), acupuncture, and ozone therapy (31). However, it is noteworthy that these analgesics may elicit adverse reactions, such as dizziness, nausea, and vomiting. Moreover, with the decline in immune function associated with aging, the incidence rate of herpes zoster in the elderly is higher (32). Given that elderly patients often receive various medications to treat chronic illnesses, the cumulative intake of painkillers amplifies the strain on liver and kidney functions, posing a significant threat to overall health. Consequently, the multifaceted nature of PHN and its treatment emphasizes the need for meticulous and holistic approaches to effectively manage pain while mitigating potential adverse effects on overall health.

BTX-A is a potent neurotoxin that can inhibit the release of the neurotransmitter acetylcholine from presynaptic neurons and regulate pain neurotransmitters (33), thereby alleviating neuropathic pain. Several meta-analyses have reported that BTX-A is effective in treating various types of neuropathic pain, including headaches, migraines, arthritic pain, cerebral palsy with acute sialadenitis, PHN, trigeminal neuralgia (TN), painful radiculopathy, diabetic neuropathy (DN), HIV-related pain, amputation, peripheral nerve injury pain, piriformis syndrome, spasticity, spinal cord injury, and intractable chronic occipital neuralgia (34). Meng et al. (12) analyzed 12 RCTs and proposed that BTX-A is safer and more effective in relieving neuropathic pain compared to saline. Shackleton et al. (35) analyzed six RCTs and reported that BTX-A was more effective than placebo for managing trigeminal neuralgia and post-herpetic neuralgia. Morra et al. (36) evaluated four RCTs on BTX-A therapy for trigeminal neuralgia, suggesting that BTX-A may be a promising and safe treatment option. However, there is limited comparative analysis of BTX-A with other treatment options for PHN. A recent study compared the efficacy of BTX-A and single nerve root pulse radiofrequency therapy (RFT) in the treatment of postherpetic neuralgia, finding similar levels of pain relief in both treatment groups (37). Our meta-analysis included 14 randomized controlled studies, demonstrating that BTX-A is a more effective option compared to analgesics.

In our meta-analysis, although BTX-A was sourced from the same manufacturer, there were variations in the injection dosage, frequency, and method. Most patients received a dose of 50–100 units, administered subcutaneously or intradermally. For patients with herpes zoster neuralgia, subcutaneous or intradermal injections are primarily used to block peripheral nerve endings. However, the impact of different injection techniques on therapeutic efficacy can vary. The characteristics of different tissues may affect the distribution and concentration of the toxin. Therefore, in addition to addressing issues of effectiveness and safety, it is crucial to further investigate the optimal use of BTX-A, This includes determining the most effective dosage, frequency of administration, and injection method.

Our meta-analysis indicates that there is no significant difference in the probability of adverse reactions between patients receiving BTX-A treatment and those receiving analgesic treatments. In addition, a subcutaneous injection of BTX-A does not have serious side effects. The observed adverse events included mild pain, dizziness, drowsiness, nausea, peripheral edema, ataxia, facial erythema, and slight muscle relaxation at the injection site. Importantly, these events are generally of mild intensity, suggesting a tolerable level of discomfort. Our findings indicate that there is no difference between a subcutaneous injection of BTX-A and analgesics in terms of safety. Consequently, there is no discernible difference in safety between the two modalities, further supporting the viability of subcutaneous BTX-A as a safe alternative in the treatment of PNH.

Our meta-analysis has certain limitations and shortcomings. First, the study design heterogeneity of the included trials is relatively high, which may be attributed to the significant differences in the baseline characteristics among the patients who received BTX-A and those who received analgesics. In addition to BTX-A or analgesics, some patients in the trial also received other treatments. Second, it is important to note that all the studies were conducted in China and that most of the studies had small sample sizes. This may have introduced potential publication bias, as evidenced by the funnel plot asymmetry and Begg’s tests. Third, although random grouping was carried out in the study, details regarding concept allocation, the blinding of participants and personal information, and the blinding of outcome assessments were not explicitly mentioned, which may have led to the risk of bias. Finally, the meta-analysis focused on short- to medium-term outcomes (up to 24 weeks) but failed to address the long-term efficacy and safety of BTX-A for PHN. This provides the next research direction, considering the chronic nature of PHN. Therefore, we suggest that future studies should employ larger-scale and higher-quality designs to further enhance the accuracy and reliability of the results.

## Conclusion

A meta-analysis of VAS results from randomized controlled trials showed that BTX-A has certain advantages in relieving postherpetic neuralgia. Furthermore, we can reasonably conclude that BTX-A is safe for treating postherpetic neuralgia, with no notable side effects. However, all the studies were conducted in Chinese patients using Chinese Botox at a dose of 100 U, and there was high heterogeneity among the patients and study designs. Additional studies should be conducted to draw definite conclusions on the efficacy of BTX-A and dosage recommendations.

## Data Availability

The raw data supporting the conclusions of this article will be made available by the authors, without undue reservation.
