# Supplementary material for: Efficacy and safety of subcutaneous injection of botulinum toxin in the treatment of Chinese postherpetic neuralgia compared to analgesics: a systematic review of randomized controlled trials and meta-analysis
Source: Front Neurol. 2024 Oct 17;15:1479931. doi: 10.3389/fneur.2024.1479931 (PMC11524963; doi:10.3389/fneur.2024.1479931)
Supplement: Supplementary file 1 [file Table_1.DOCX]

**Search strategy**

Pubmed

#1 ((((((((((((((((((herpes zoster neuralgia[MeSH]) OR (Ramsay Hunt Auricular Syndrome[Title/Abstract])) OR (Neuralgia[Title/Abstract])) OR (Geniculate[Title/Abstract])) OR (Geniculate Neuralgia[Title/Abstract])) OR (Geniculate Neuralgias[Title/Abstract])) OR (Neuralgias, Geniculate[Title/Abstract])) OR (Ramsay Hunt Syndrome[Title/Abstract])) OR (Syndrome, Ramsay Hunt[Title/Abstract])) OR (Auricular Syndrome of Ramsay Hunt[Title/Abstract])) OR (Herpes Zoster Cephalicus[Title/Abstract])) OR (Herpetic Geniculate Ganglionitis[Title/Abstract])) OR (Ganglionitis, Herpetic Geniculate[Title/Abstract])) OR (Geniculate Ganglionitides, Herpetic[Title/Abstract])) OR (Geniculate Ganglionitis, Herpetic[Title/Abstract])) OR (Herpetic Geniculate Ganglionitides[Title/Abstract])) OR (Herpes Zoster Auricularis[Title/Abstract])) OR (Geniculate Herpes Zoster[Title/Abstract])) OR (Herpes Zoster, Geniculate[Title/Abstract])

#2 (((((((((((Botulinum toxin[MeSH]) OR (Toxins, Botulinum[Title/Abstract])) OR (Botulinum Neurotoxins[Title/Abstract])) OR (Neurotoxins, Botulinum[Title/Abstract])) OR (Botulinum Toxin[Title/Abstract])) OR (Toxin, Botulinum[Title/Abstract])) OR (Clostridium[Title/Abstract])) OR (botulinum Toxins[Title/Abstract])) OR (Toxins, Clostridium botulinum[Title/Abstract])) OR (Botulinum Neurotoxin[Title/Abstract])) OR (Neurotoxin, Botulinum[Title/Abstract])) OR (Botulin[Title/Abstract])

#3 (((((((randomized controlled trial[Title/Abstract]) OR (randomized[Title/Abstract])) OR (controlled clinical trial[Title/Abstract])) OR (controlled[Title/Abstract])) OR (placebo[Title/Abstract])) OR (trial[Title/Abstract])) OR (random[Title/Abstract])) OR (groups[Title/Abstract])

#1 and #2 and #3

Web of Science

TS=(Herpes Zoster Neuralgia OR Ramsay Hunt Auricular Syndrome OR Neuralgia OR Geniculate OR Geniculate Neuralgia OR Geniculate Neuralgias OR Neuralgias, Geniculate OR Ramsay Hunt Syndrome OR Syndrome, Ramsay Hunt OR Auricular Syndrome of Ramsay Hunt OR Herpes Zoster Cephalicus OR Herpetic Geniculate Ganglionitis OR Ganglionitis, Herpetic Geniculate OR Geniculate Ganglionitides, Herpetic OR Geniculate Ganglionitis, Herpetic OR Herpetic Geniculate Ganglionitides OR Herpes Zoster Auricularis OR Geniculate Herpes Zoster OR Herpes Zoster, Geniculate) AND TS= (Botulinum toxin OR Toxins, Botulinum OR Botulinum Neurotoxins OR Neurotoxins, Botulinum OR Botulinum Toxin OR Toxin, Botulinum OR Clostridium OR botulinum Toxins OR Toxins, Clostridium botulinum OR Botulinum Neurotoxin OR Neurotoxin, Botulinum OR Botulin) AND TS= (randomized controlled trial OR randomized OR controlled clinical trial OR plaecbo OR controlled OR trial OR randomly OR groups)

Cochrane Library

(Herpes Zoster Neuralgia OR Ramsay Hunt Auricular Syndrome OR Neuralgia OR Geniculate OR Geniculate Neuralgia OR Geniculate Neuralgias OR Neuralgias, Geniculate OR Ramsay Hunt Syndrome OR Syndrome, Ramsay Hunt OR Auricular Syndrome of Ramsay Hunt OR Herpes Zoster Cephalicus OR Herpetic Geniculate Ganglionitis OR Ganglionitis, Herpetic Geniculate OR Geniculate Ganglionitides, Herpetic OR Geniculate Ganglionitis, Herpetic OR Herpetic Geniculate Ganglionitides OR Herpes Zoster Auricularis OR Geniculate Herpes Zoster OR Herpes Zoster, Geniculate):ti,ab,kw AND (Botulinum toxin OR Toxins, Botulinum OR Botulinum Neurotoxins OR Neurotoxins, Botulinum OR Botulinum Toxin OR Toxin, Botulinum OR Clostridium OR botulinum Toxins OR Toxins, Clostridium botulinum OR Botulinum Neurotoxin OR Neurotoxin, Botulinum OR Botulin):ti,ab,kw AND (randomized controlled trial OR randomized OR controlled clinical trial OR plaecbo OR controlled OR trial OR randomly OR groups):ti,ab,kw

Embase

#1.'herpes zoster neuralgia'/exp OR 'herpes zoster neuralgia' OR 'ramsay hunt auricular syndrome' OR 'neuralgia'/exp OR 'neuralgia' OR 'geniculate' OR 'geniculate neuralgia'/exp OR 'geniculate neuralgia' OR 'geniculate neuralgias' OR 'neuralgias, geniculate' OR 'ramsay hunt syndrome'/exp OR 'ramsay hunt syndrome' OR 'syndrome, ramsay hunt' OR 'auricular syndrome of ramsay hunt' OR 'herpes zoster cephalicus'/exp OR 'herpes zoster cephalicus' OR 'herpetic geniculate ganglionitis'/exp OR 'herpetic geniculate ganglionitis' OR 'ganglionitis, herpetic geniculate' OR 'geniculate ganglionitides, herpetic' OR 'geniculate ganglionitis, herpetic' OR 'herpetic geniculate ganglionitides' OR 'herpes zoster auricularis'/exp OR 'herpes zoster auricularis' OR 'geniculate herpes zoster'/exp OR 'geniculate herpes zoster' OR 'herpes zoster, geniculate'

#2.'Botulinum toxin’ OR ‘Toxins, Botulinum’ OR ‘Botulinum Neurotoxins’ OR ‘Neurotoxins, Botulinum’ OR ‘Botulinum Toxin’ OR ‘Toxin, Botulinum’ OR ‘Clostridium’ OR ‘botulinum Toxins’ OR ‘Toxins, Clostridium botulinum' OR 'Botulinum Neurotoxin' OR ’Neurotoxin, Botulinum' OR 'Botulin'

#3.'randomized controlled trial (topic)'/exp OR randomized:ab,ti OR 'controlled clinical trial':ab,ti OR controlled:ab,ti OR placebo:ab,ti OR trial:ab,ti OR randomly:ab,ti OR groups:ab,ti

#1 and #2 and #3

CNKI and Wanfang

(带状疱疹性神经痛 or 带状疱疹后遗神经痛 or 带状疱疹) and (肉毒毒素 or 肉毒杆菌)
